# The Relationship Between Parental Phubbing and Preschoolers’ Behavioral Problems: The Mediation Role of Mindful Attention Awareness

**DOI:** 10.3390/children12081022

**Published:** 2025-08-02

**Authors:** Antonio Puligheddu, Annamaria Porru, Andrea Spano, Stefania Cataudella, Maria Lidia Mascia, Dolores Rollo, Cristina Cabras, Maria Pietronilla Penna, Daniela Lucangeli

**Affiliations:** 1 Department of Developmental Psychology and Socialization, University of Padova, Via Venezia, 8, 35131 Padova, PD, Italy; antonio.puligheddu@unipd.it (A.P.); daniela.lucangeli@unipd.it (D.L.); 2 Department of Pedagogy, Psychology, Philosophy, University of Cagliari, Via Is Mirrionis 1, 09123 Cagliari, CA, Italy; andrea.spano88@unica.it (A.S.); scataudel@unica.it (S.C.); ccabras@unica.it (C.C.); penna@unica.it (M.P.P.); 3 Department of History, Human Sciences and Education, University of Sassari, Via Maurizio Zanfarino 62, 07100 Sassari, SS, Italy; mlmascia@uniss.it; 4 Department of Medicine and Surgery, University of Parma, Via Volturno, 39, 43125 Parma, PR, Italy; dolores.rollo@unipr.it

**Keywords:** parental phubbing, parental mindfulness, preschoolers, behaviors

## Abstract

Phubbing, a relatively new phenomenon in the field of digital risks, refers to the act of ignoring someone in favor of focusing on a smartphone during face-to-face interactions. Parental phubbing, a specific form of this behavior, is a prevalent negative parenting practice that can affect parent–child relationships and child development. However, the impact of parental phubbing on the emotional and behavioral development of preschool children remains unclear. This study aims to explore the relationship between parental phubbing and preschoolers’ behavioral problems, as well as test whether parents’ mindful attention awareness (MAA) acts as a mediator between them. Method: A questionnaire was administered to 138 Italian parents (mean age = 38.5, SD = 6.2) of 138 kindergarten preschoolers (mean age = 3.9, SD = 1.03). Questionnaires included the Generic Scale of Phubbing (GSP), the Mindful Attention Awareness Scale (MAAS), and the Strengths and Difficulties Questionnaire (SDQ). Results: Analyses revealed a significant negative correlation between the MAAS and SDQ total scores, a positive correlation between the GSP total score and the SDQ total score, and a negative correlation between the GSP total score and the MAAS total score. The mediation analysis did not show a direct effect of GSP on SDQ, suggesting that parental phubbing did not directly predict children’s behavioral difficulties. Nevertheless, the indirect effect measured by bootstrapping was significant, indicating that parental MAA fully mediated the relationship between parental phubbing and preschoolers’ problematic behaviors. Conclusions: Although further research is needed, parental mindfulness may influence phubbing behaviors in parents providing valuable insights for early interventions aimed at reducing problem behaviors in young children.

## 1. Introduction

Smartphones have become an essential part of many people’s lives. The widespread use of smartphones for everyday activities has led to the phenomenon of ‘phubbing’, a portmanteau of ‘phone’ and ‘snubbing’, referring to the act of ignoring someone to focus on the smartphone during face-to-face interactions [1,2,3]. Many studies highlight the negative impact of phubbing in workplaces [4], romantic relationships [5], and education [6]. However, the effects of phubbing on young children’s emotional and behavioral issues are less understood. Recently, the occurrence of phubbing within the family setting has gained attention as a significant social concern. Parental phubbing is a prevalent negative parenting practice that affects parent–child relationships and child development [7,8,9]. Studies increasingly examine how parental phubbing undermines emotional connections between parents and children, leading to reduced parental sensitivity, responsiveness [10] and feelings of closeness [11]. Research shows that parents who frequently engage in phubbing tend to exhibit higher levels of smartphone overuse (sometimes addiction) and are more likely to adopt authoritarian parenting styles [12]. These parents often demonstrate fewer positive postures, such as listening and warmth, relying more on authoritarian behaviors, such as commands, threats, and deprivations [13]. These attitudes can result in children’s emotional and behavioral issues [13,14] as well as in increased likelihood of engaging in risky behaviors, which elevates the risk of injury, particularly in younger children [15,16,17,18,19]. Children exposed to parental phubbing have also been found to struggle with delaying gratification, an effect mediated by both the child’s and mother’s positive emotions [20].

Moreover, the association of phubbing with several developmental challenges of children [14,21,22,23,24,25] highlights the need for interventions that address this growing problem.

### 1.1. Relationship of Parental Phubbing and Mindful Attention Awareness (MAA)

Since parental phubbing reflects a lack of physical and mental engagement during interaction with children [24], it can be interpreted as a parenting behavior characterized by low levels of interpersonal mindfulness during parent–child interactions. Mindfulness is defined as the intentional focus on the present moment with an open, non-judgmental, and non-reactive attitude [26], characterized by curiosity and attentiveness to the here and now [27].

In today’s digital age, in which adults extensively use smartphones in daily life, being engaged simultaneously in both the physical and digital environments via smartphones is often criticized for distracting attention, fostering dependency, and diminishing the quality of in-person interactions [28]. Even when they are not actively used, smartphones’ mere presence can cause distractions [29]. Furthermore, frequent thoughts about one’s smartphone [30] and staring at screens can make conversations less fulfilling [31].

In this context, parental phubbing can be viewed as a negative form of parenting, marked by low interpersonal mindfulness. When parents are engrossed in their smartphones, they may unintentionally neglect their children’s basic needs, leading to feelings of social exclusion and a reduced sense of belonging in those who are phubbed [2,32]. Phubbing limits eye contact and engagement in conversations, causing individuals to become less attentive and experience divided attention [33,34]. Consequently, parents who are less mindful may be more prone to smartphone addiction and phubbing.

Research suggests a negative correlation between mindfulness and smartphone addiction in adults [35,36]. Excessive smartphone use has been linked to reduced focus and productivity [37], while mindfulness can mitigate problematic smartphone use by reducing risks such as nomophobia and boredom [38], improving self-control [39], and enhancing self-regulation [40]. Mindfulness has also been used to treat addictive behaviors, including automatic phone-checking [41,42]. Thus, it can be expected that mindfulness acts as a protective factor against excessive smartphone use in adults, including parents.

Although there are few studies on mindfulness and phubbing, the available research shows an inverse relationship between them [43,44]. When people are less mindful of others and their own actions, they are more likely to engage in phubbing [44].

Several studies have reported a link between mindfulness and phubbing, revealing that relationship satisfaction and perceived relationship quality decrease in adults exposed to their partner’s phubbing behavior. Furthermore, this decrease negatively impacts overall life satisfaction [45]

Therefore, it can be inferred that mindfulness may increase the awareness of their habits, helping individuals regain control over their smartphone use and reduce phubbing behavior, particularly among parents.

### 1.2. Relationship of Phubbing and Externalizing and Internalizing Problems

In previous studies, several negative outcomes for children who experience parental phubbing are highlighted. For instance, parents who frequently use their phones while interacting with their children report more behavioral problems in their children [14].

Two typical types of behaviors are recognized as significant predictors of the social competence of children: externalizing dysregulated behaviors such as aggression, impulsivity, and control problems and internalizing dysregulated behaviors such as withdrawal, anxiety, and depression [46,47,48].

Previous research [49] has shown that parenting styles affect infants’ and toddlers’ social, emotional, and intellectual development.

Parents who engage in phubbing tend to display negative parenting postures, including neglect, impatience, and a lack of warmth, which can contribute to behavioral issues in their children [13]. Moreover, adolescents whose parents regularly prioritize phone use over a direct interaction with them are more likely to engage in harmful activities like online bullying [50]. These negative consequences are strongly linked to heightened anxiety, poor self-control, and other emotional challenges in both children and adolescents [51].

However, most research on parental phubbing focuses on its effects on adolescents rather than younger children [13,52], leaving this field underexplored. Few exceptions concern the studies aimed to examine how parental phubbing affects children’s behavior [14,23,24,53], making it important to investigate its influence on both internalizing and externalizing behaviors. Internalizing behaviors involve children’s self-isolation and their difficulties with social situations, which can lead to developmental issues [54,55]. Externalizing behaviors refer to intentionally harming others or violating social norms, resulting in negative effects on children’s emotional and physical well-being, and often lead to poor social relationships [56,57]. Understanding how parental phubbing contributes to these issues is vital for addressing its impact on child development.

Indeed, parents who are distracted by their phones during interactions with their children often fail to respond with emotional warmth to children’s needs [14]. In addition, being phubbed by their parents makes children more likely to develop behavioral problems, including aggression and social withdrawal [58]. Furthermore, such distractions can interfere with the development of prosocial behaviors, like helping and sharing, which are necessary to counterbalance problem behaviors [59]. Gender differences can also emerge, with more peer problems in boys and reduced prosocial behavior in girls [60,61].

Since children learn about the world through their interactions with their parents [62], when these interactions are disrupted by technology the consequences can be far-reaching. Parental phubbing not only weakens emotional bonds but may also reduce prosocial behaviors, and increase internalizing and externalizing behaviors [14,53]. Understanding these dynamics highlights the importance of minimizing distractions during parent–child interactions to foster healthier emotional and behavioral development in children.

### 1.3. Relationship of MAA and Externalizing and Internalizing Problems

According to Duncan et al. [63], mindful parenting consists of attentive listening, non-judgmental acceptance, emotional awareness, self-regulation, and compassion, both for the parent and for the child. Therefore, mindful parenting allegedly has a positive influence on general parenting, parental well-being, child management practices, and parent–child affection, which in turn reduce youth problem outcomes and increase youth positive outcomes [63]. This is also shown in several intervention studies that targeted mindful parenting. Studies provided by Coatsworth et al. [64,65] demonstrates that improvements in mindful parenting positively influence child management practices and parent–youth relationships. A positive impact of mindful parenting on the relationship between parents and children and subsequently on the children’s well-being is also shown by Medeiros et al. [66]. Similarly, in a literature review about mindful parenting and mindfulness interventions aimed at parents, Bröning and Brandt [67] reported positive effects of mindful parenting for the well-being of parents and children through parental self-regulation and positive parent–child-interactions. These favorable effects seem to also carry over to children’s behavior, as demonstrated in a mindfulness-based stress reduction (MBSR) intervention study for parents of children with developmental delays [68]. This study reports that the improved well-being of participating parents is also reflected in less behavior problems from their children. In another mindfulness intervention study with parents of children with developmental disabilities, Singh et al. [69] found positive outcomes for parents and positive behavior change in children, especially concerning aggressive and prosocial behaviors. Apart from improvements in the parent–child-relationship, parental self-regulation, and well-being, mindfulness in parenting can also help to reduce family conflict [70] and improve mother–child communication and adolescent self-disclosure [71], which in turn may decrease internalizing and externalizing problems of children. Indeed, several studies demonstrate a negative relationship between mindful parenting and internalizing and externalizing problems in children and adolescents [67]. In a scoping review of 16 studies concerning mindful parenting training for parents of young children with behavioral problems, Donovan et al. [72] concluded that mindfulness interventions can improve child behavior with a small effect size. This is also confirmed in a systematic review of seven randomized controlled trials by Townshend et al. [73], who found specific evidence that mindful parenting programs can increase positive parenting and reduce preschooler’s externalizing symptoms. This also applies to mindfulness programs for children and adolescents. In a mindfulness intervention study with adolescents affected by different externalizing disorders, Bögels et al. [74] showed that the participants of a mindfulness training reported less internalizing and externalizing complaints after the intervention period compared to the waitlist period before starting the intervention. These symptom improvements were also confirmed by their parents. In another, cross-sectional study including adolescents and their parents, only non-judgmental acceptance of parental functioning as a dimension of mindful parenting is significantly related to adolescent-reported internalizing problems, namely depression and anxiety [75]. This implies that specific domains of mindful parenting might be especially relevant to specific aspects of behavioral problems in children and adolescents. Taken together, mindfulness in parenting may help to reduce internalizing and externalizing problems of children through different mechanisms, including improvements in the parent–child relationship, parent–child communication, and well-being of parents and children.

Another study showed that parental phubbing mediated the link between parenting MAA and children’s externalizing behaviors [76] and executive function [77]. Several studies have reported a link between mindfulness and phubbing, revealing that a high level of mindful parenting is associated with less parental phubbing as reported by parents [76].

In summary, these findings suggest that the direct associations between phubbing and mindfulness vary depending on the context and that mindfulness plays a moderating and mediating role in phubbing-related behavioral pathways.

### 1.4. The Present Study

However, the existing literature offers limited and sometimes inconsistent evidence regarding the mechanisms through which parental smartphone use—particularly phubbing—is associated with young children’s emotional and behavioral development, especially in relation to parental MAA as a potential protective factor.

The purpose of this study was to explore how parental phubbing behavior and mindfulness impact problematic behavior in preschool-aged children. We hypothesize that (1) parental mindfulness (MAA) will be negatively associated with children’s behavioral problems, and (2) parental phubbing will be positively associated with those problems. Therefore, we also expect to find (3) a negative correlation between parental phubbing and mindfulness. Additionally, this study investigates whether mindfulness in parents serves as a mediator: (4) we propose a mediation model in which parental mindfulness mediates the effect of parental phubbing on behavioral problems in preschoolers. According to this model, parental mindfulness is expected to reduce both phubbing behaviors and behavioral difficulties in children, thus accounting for the indirect pathway between the two.

## 2. Materials and Methods

### 2.1. Participants

A total of 138 Italian parents (mean age = 38.06, SD = 6.12; 121 mothers and 15 fathers) filled out self-report and parent-report questionnaires concerning a total of 138 children (mean age = 3.9, SD = 1.03; 67 girls and 62 boys, 9 did not disclose gender). A total of 157 parents completed the questionnaire, including instances where both parents responded for the same child. In this case, to avoid the partial duplication of data, 19 parents were excluded from the analysis.

With a sample size of 138 participants, we calculated the post hoc statistical power of the observed correlation between the variable used for the mediation model using a Monte Carlo simulation approach specifically designed for mediation analysis [78]. We conducted the simulation via the online Monte Carlo power calculator for indirect effects, entering the correlations (M-X = −0.35, Y-X = 0.19, Y m = −0.36; M = MAAS; X = GSP; Y = SDQ) and standard deviations (X = 8.63, M = 9.58, Y = 3.63), with a sample size of N = 138. The resulting estimated power was approximately 96%. This exceeds the commonly accepted threshold of 80%, suggesting that the analysis was sufficiently powered. Parents answered questions about their own and their partner’s educational background and profession. The responses concerning education and profession were converted into numbers and averaged between both parents to reflect the total socioeconomic status of the couple. Averaged between partners, most couples completed secondary school (31.17%) or pre-university courses (29.87%) and worked in specialized work or farming (25.81%) or pursued executive professions or office work (23.23%). This study was approved by the Ethics Committee of the University of Cagliari (protocol number: 0000611, issued on 04 January 2024) and performed according to the principles expressed in the 1964 Declaration of Helsinki.

### 2.2. Procedure

We conducted a cross-sectional quantitative study, with non-random convenience sampling of parents. By means of an online survey, we assessed parental phubbing, parental mindfulness, and internalizing and externalizing difficulties of children. The sample was recruited by sending an email to the preschools. The purposes of this research reported in the informed consent were explained in the email. We contacted the kindergarten directors to explain our research intentions and received their support. In addition, we provided instructions to the kindergarten parents and obtained their consent to participate in the study by anonymously filling out the questionnaire.

The survey was administered in Italian via the online Qualtrics platform. The system prevented missing data by requiring participants to complete all items before submission. Completion of the questionnaire took between 10 and 15 min.

Data collection took place between February 2024 and June 2024, and throughout that period, the online survey was permanently accessible.

### 2.3. Measures

#### 2.3.1. Parental Phubbing

Parental phubbing was assessed using an Italian translation of the Generic Scale of Phubbing (GSP) [79], which consists of 15 items across four dimensions: nomophobia (e.g., I feel anxious if my phone is not nearby; 4 items; α = 0.84); interpersonal conflict (e.g., I have conflicts with others because I am using my phone; 4 items; α = 0.87); self-isolation (e.g., I would rather pay attention to my phone than talk to others; 4 items; α = 0.83); and problem acknowledgement (e.g., I pay attention to my phone for longer than I intend to do so; 3 items; α = 0.82). Participants responded to each item on a 7-point Likert scale (1 = never, 2 = rarely, 3 = occasionally, 4 = sometimes, 5 = frequently, 6 = usually, 7 = always). Subscale scores were computed, and an overall phubbing score (α = 0.93) was obtained by summing the subscale scores, with higher scores indicating greater levels of phubbing behavior.

#### 2.3.2. Parental Mindful Attention Awareness

Parental mindfulness was assessed using the unidimensional Mindful Attention Awareness Scale (MAAS) developed by Brown and Ryan [80], which measures individual differences in the absence of attention and awareness to the present moment. The scale consists of 15 items rated on a 6-point Likert scale (1 = almost always, 2 = very frequently, 3 = somewhat frequently, 4 = somewhat infrequently, 5 = very infrequently, and 6 = almost never), with participants indicating how often they experience states of mindlessness in daily life. An overall mindfulness score is calculated by averaging the responses across all items, with higher scores indicating greater mindfulness. For the present study, we used the Italian items from the adaptation by Veneziani and Voci [81], along with an Italian translation of the original response scale by Brown and Ryan [80]. The translated items have shown good psychometric properties (α = 0.84), a unidimensional factor structure and divergent and convergent validity [81].

#### 2.3.3. Internalizing and Externalizing Difficulties of Children

Children’s internalizing and externalizing difficulties were assessed using the Strengths and Difficulties Questionnaire (SDQ), developed by Goodman [82] as a revision of the Rutter parent questionnaire [83], with the addition of items focusing on children’s strengths. The questionnaire consists of 25 items divided into five subscales: conduct problems (e.g., Often has temper tantrums or hot tempers; 5 items; α = 0.84), emotional symptoms (e.g., Often unhappy, down-hearted or tearful; 5 items; α = 0.75), hyperactivity–inattention (e.g., Restless, overactive, cannot stay still for long; 5 items; α = 0.86), peer problems (e.g., Rather solitary, tends to play alone; 5 items; α = 0.68), and prosocial behavior (e.g., Considerate of other people’s feelings; 5 items; α = 0.83) [84]. Items are rated on a 3-point Likert scale (0 = not true, 1 = somewhat true, 2 = certainly true), and subscale scores are computed by summing the items after reverse-coding where necessary.

The total difficulties score (α = 0.88) is derived by summing all subscales except prosocial behavior, yielding a maximum score of 40, with higher scores indicating greater difficulties related to behavior, emotions, and peer relationships [84].

In this study, the Italian adaptation of the SDQ by Marzocchi et al. [85] was used.

### 2.4. Data Analyses

To test our hypothesis, we first checked the parents’ data for multivariate outliers in the total scores and subscales, using Mahalanobis distances with a threshold of 0.975. After the exclusion of multivariate outliers, we tested the univariate normal distribution of the total scores, using Shapiro–Wilks tests. For descriptive statistics, the means and standard deviations of the GSP, MAAS, and SDQ total scores were calculated. To understand how parental phubbing, parental mindfulness, and internalizing and externalizing difficulties of children are related, we calculated Pearson’s correlations with all the subscales and total scores from the parents’ data while filtering out parent’s age and children’s age as potential confounding variables. Moreover, we calculated Pearson’s correlations between the parents’ and teachers’ SDQ ratings.

Afterwards, we tested a mediation model with the GSP total score as a predictor, the MAAS total score as a mediator, and the SDQ total score as the outcome, using children’s age as a control variable. The mediation analysis was performed using a bootstrapping technique with 1000 resamples. The data analysis was conducted in JASP JASP Team (2024). JASP (Version 0.19.3) [Computer software] [86] and RStudio [Version 2023.03.1 Build 446; R Development Core Team, 2016] [87].

No missing data were present in the final dataset. Partial responses (e.g., incomplete questionnaires) were not recorded by the system and therefore were not included in the dataset.

## 3. Results

The sample was checked for univariate normal distribution, which could not be confirmed for the GSP total score (W = 0.93, *p* = <0.001) and neither for the MAAS total score (W = 0.98, *p* = 0.033) and the SDQ total score (W = 0.97, *p* = 0.004). Parent-rated GSP, MAAS, and SDQ had average total scores of M = 27.61 (SD = 9.08), M = 4.59 (SD = 0.66), and M = 7.79 (SD = 3.66), respectively. Since Pearson’s correlation is robust in samples larger than 30 [81], it was calculated despite the total scores of GSP, MAAS, and SDQ not being normally distributed.

**Figure 1:**
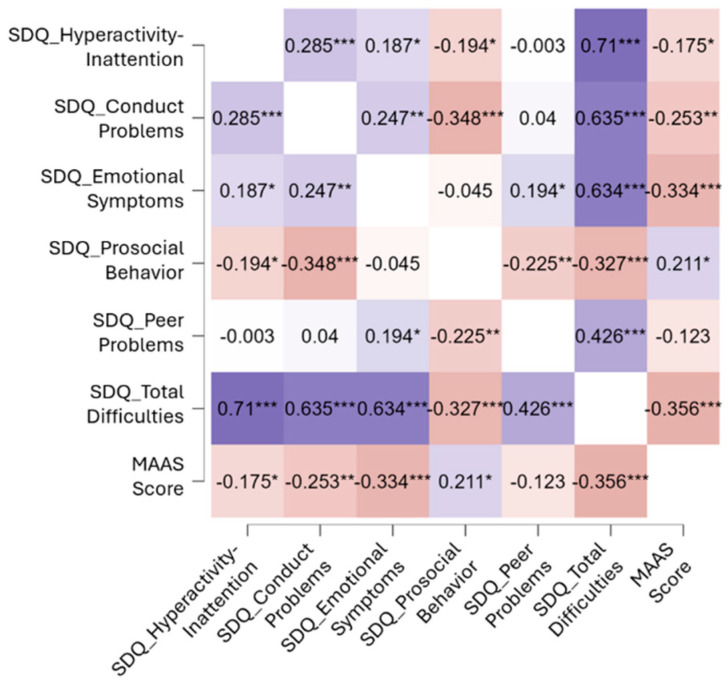
Correlation analysis between MAAS (Mindful Attention Awareness Scale) and SDQ (Strengths and Difficulties Questionnaire). The figure displays Pearson’s correlation coefficients between SDQ subscales (hyperactivity–inattention, conduct problems, emotional symptoms, prosocial behavior, peer problems, and total difficulties) and parental mindfulness (MAAS). Warmer shades represent negative correlations, whereas cooler shades indicate positive correlations. Asterisks denote significance levels (* = *p* < 0.05, ** = *p* < 0.01, *** = *p* < 0.001).

In relation to our first hypothesis, we examined whether children’s behavioral problems were associated with parental mindfulness, hypothesizing a negative correlation between these two variables. Our analysis revealed a significant negative correlation between the MAAS and SDQ total scores (r = −0.356, *p* < 0.001) (see Figure 1), supporting our hypothesis. Furthermore, the MAAS total score demonstrated significant negative correlations with SDQ hyperactivity–inattention (r = −0.175, *p* = 0.044), SDQ conduct problems (r = −0.253, *p* = 0.003), and SDQ emotional difficulties (r = −0.334, *p* < 0.001), as well as a significant positive correlation with SDQ prosocial behavior (r = 0.211, *p* = 0.015).

**Figure 2:**
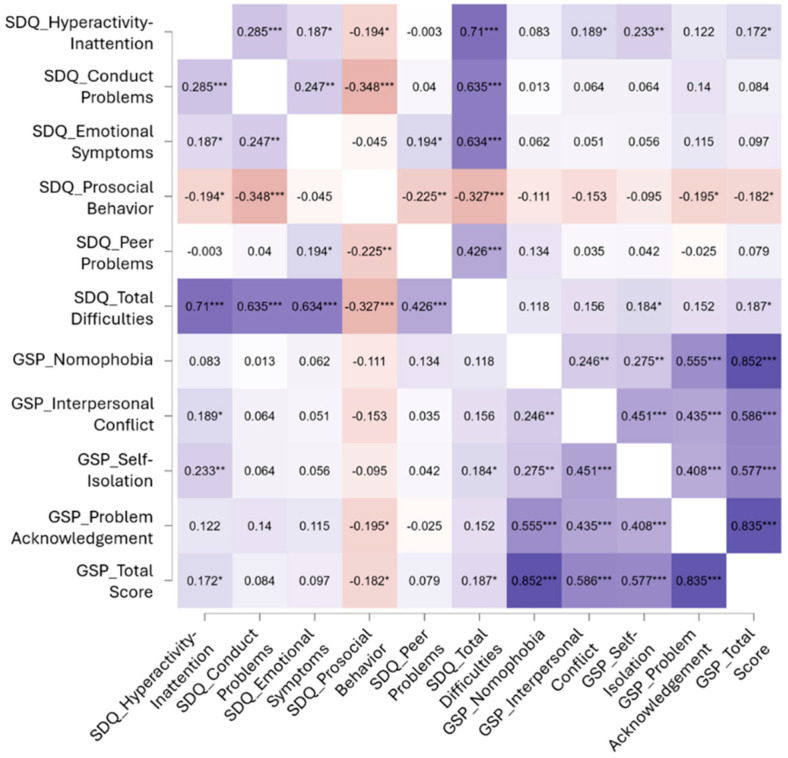
Correlation analysis between GSP (Generic Scale of Phubbing) and SDQ (Strengths and Difficulties Questionnaire). The figure displays Pearson’s correlation coefficients between SDQ subscales (hyperactivity–inattention, conduct problems, emotional symptoms, prosocial behavior, peer problems, and total difficulties) and the components of the Generic Scale of Phubbing (GSP; nomophobia, interpersonal conflict, self-isolation, problem acknowledgement, and total score). Warmer shades represent negative correlations, whereas cooler shades indicate positive correlations. The intensity of the color corresponds to the strength of the association. Asterisks denote significance levels (* = *p* < 0.05, ** = *p* < 0.01, *** = *p* < 0.001).

For our second research question, we investigated whether parental phubbing was related to children’s behavioral difficulties. We hypothesized that greater behavioral problems in children would be associated with higher levels of parental phubbing. Consistent with this hypothesis, correlational analyses revealed a positive correlation between the GSP total score and the SDQ total score (r = 0.187, *p* = 0.031) (see Figure 2). Additionally, significant correlations were observed between the SDQ subscales and the GSP subscales, specifically between SDQ hyperactivity–inattention and GSP self-isolation (r = 0.233, *p* = 0.007) and SDQ prosocial behavior and GSP problem acknowledgment (r = −0.195, *p* = 0.024).

**Figure 3:**
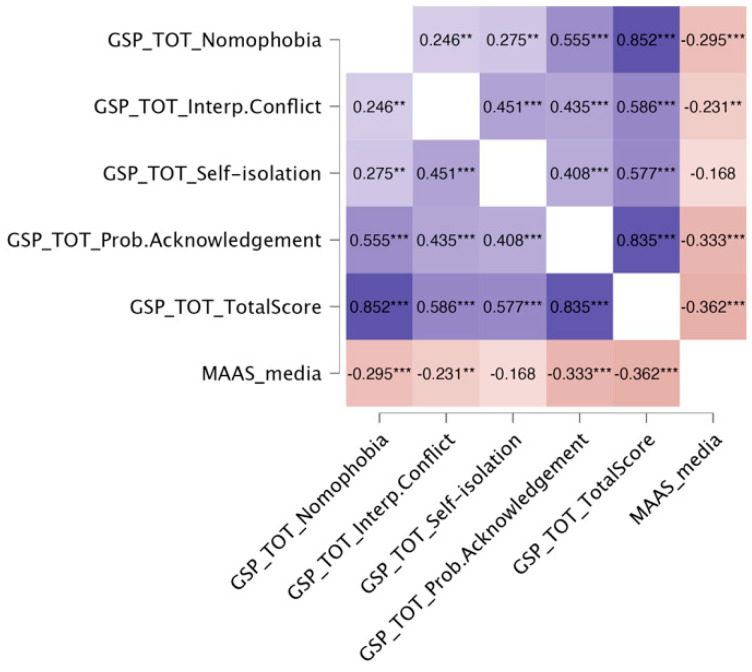
Correlation analysis between GSP (Generic Scale of Phubbing) and MAAS (Mindful Attention Awareness Scale). The figure displays pairwise Pearson’s correlation coefficients among SDQ subscales (hyperactivity–inattention, conduct problems, emotional symptoms, prosocial behavior, peer problems, and total difficulties), the components of the Generic Scale of Phubbing (GSP: nomophobia, interpersonal conflict, self-isolation, problem acknowledgement, and total score), and parental mindfulness (MAAS). Cooler shades (blue) indicate positive correlations, whereas warmer shades (red) represent negative correlations. The intensity of the color reflects the magnitude of the correlation. Asterisks denote significance levels (** = *p* < 0.01, *** = *p* < 0.001).

Next, we examined the relationship between parental mindfulness and phubbing, hypothesizing a negative correlation. Our findings showed that the GSP total score was negatively correlated with the MAAS score (r = −0.362, *p* < 0.001) (see Figure 3).

Lastly, we examined the role of mindfulness as a mediator between parental phubbing and children’s problem behaviors. In order to examine whether parental mindfulness mediated the relationship between parental phubbing and behavioral problems in preschool children, we tested a mediation model with parental mindfulness (MAAS) as the mediator, parental phubbing (GSP) as the predictor, and behavioral difficulties in children (SDQ) as the outcome.

**Figure 4:**
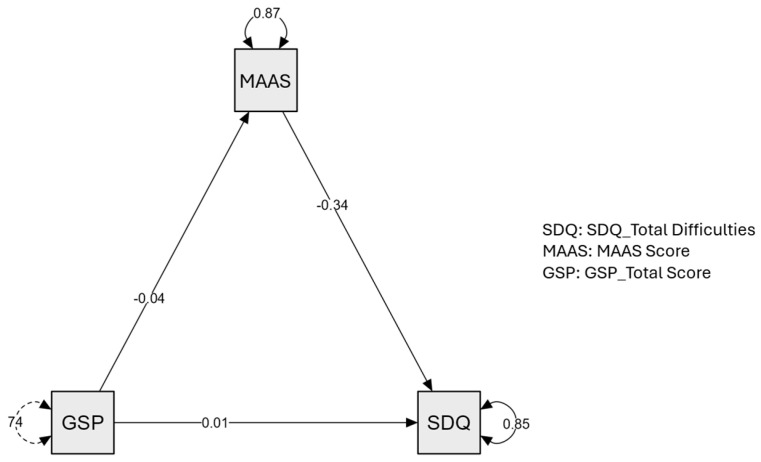
Mediation analysis between SDQ (Strengths and Difficulties Questionnaire), MAAS (Mindful Attention Awareness Scale) and GSP (Generic Scale of Phubbing). Arrows represent standardized path coefficients, with curved arrows indicating explained variance (R^2^) for each endogenous variable.

The total effect of parental phubbing on children’s behavioral problems was statistically significant (β = 0.023, SE = 0.010, z = 2.350, *p* = 0.019, 95% CI [0.001, 0.044]) (see Figure 4), indicating that higher levels of parental phubbing were associated with greater behavioral difficulties in children. However, the direct effect of parental phubbing on behavioral problems was not significant when parental mindfulness was included in the model (β = 0.009, SE = 0.010, z = 0.883, *p* = 0.377, 95% CI [–0.012, 0.031]).

Conversely, the indirect effect via parental mindfulness was significant: β = 0.014, SE = 0.005, z = 2.997, *p* = 0.003, 95% CI [0.006, 0.026]. Specifically, greater parental phubbing was associated with lower levels of parental mindfulness (β = −0.041, SE = 0.009, z = −4.489, *p* < 0.001), which in turn predicted higher levels of behavioral problems in children (β = −0.340, SE = 0.085, z = −4.024, *p* < 0.001).

Taken together, these findings support the hypothesized mediation model, suggesting that the effect of parental phubbing on children’s behavioral problems operates indirectly through reduced parental mindfulness.

## 4. Discussion

The rise of phubbing in everyday parent–child interactions has drawn attention from researchers due to its negative impact on child development. Identifying the factors that can reduce parental phubbing is essential for promoting a healthy development in children. This study aims to explore the connection between parental phubbing, parental mindfulness, and preschool children’s behavioral difficulties, as well as determine whether a mindful attitude in parents serves as a mediator in the relationship between parental phubbing and children’s problem behaviors.

Our findings revealed that mindful parents reported fewer behavioral issues in their children. Specifically, parental mindfulness was found to be negatively related to externalizing behaviors, such as hyperactivity and conduct problems, as well as internalizing behaviors like emotional difficulties in children. This aligns with earlier research showing that mindful parenting supports positive psychosocial outcomes in children and adolescents [69,75,88,89,90]. For example, Parent et al. [89] noted that higher levels of mindfulness in parents were linked to lower levels of both internalizing and externalizing issues in their children. Likewise, other studies pointed to a connection between mindful parenting and better adjustment along with fewer externalizing behavioral problems [71,91]. Conversely, a positive relationship was identified between parental mindfulness and prosocial behavior in children. This outcome may stem from the idea that mindful caregivers are more likely to offer the emotional support and security their children require, fostering trust and emotional well-being. As a result, children may become more inclined to form positive, prosocial connections with others [92].

Furthermore, phubbing behavior in parents was found to be positively associated with behavioral children’s problems. These results are in line with other studies that underline the relationship between the interference of technology in parent–child interaction and children’s behavioral difficulties [76], both internalized and externalized [13,76,93,94]. In particular, the self-isolation dimension of phubbing was found to be positively associated with hyperactivity and attention difficulties in children, confirming the results of other studies that support the relationship between parent technoference and child externalizing behavior [14,58,95]. For instance, Carson and Kuzik [95] found that higher parent–child technology interference was associated with lower executive functions (i.e., response inhibition) and self-regulation (i.e., emotional) as well as higher behavioral problems (i.e., externalizing and internalizing). Preschool years are a critical time for children to learn social skills, and if parents are constantly looking down at their phones, they may not be able to provide their children with enough opportunities for social interaction [96]. This may result in children having difficulty understanding and responding to the emotions of others, reducing their level of prosocial interaction with peers [97].

Parental phubbing behavior was also associated with lower levels of mindfulness in parents. This is congruent with previous research that pointed out a negative relationship between phubbing and mindfulness [43,44], highlighting how risk factors for problematic smartphone use can show a reduction as mindful levels increase [36].

Our findings also revealed that parental mindfulness plays a mediating role in the link between parental phubbing and children’s problematic behaviors. This mediation can be understood by recognizing that parents who engage in phubbing behaviors with their children are more likely to exhibit negative parenting practices, such as being less attentive, lacking warmth, and neglecting the child’s basic needs, either consciously or unconsciously [13,32]. Consequently, children may develop maladaptive behaviors in an effort to capture their caregivers’ attention [58]. A mindful attitude in parents may encourage healthier parenting practices, such as actively listening without judgment and responding with thoughtful, compassionate choices instead of automatic reactions [63,91,98,99], which can reduce phubbing behaviors. Furthermore, mindful parents are more aware of the harmful effects that phubbing can have on their child’s development [88,100,101] and are better able to avoid engaging in such behavior. Nevertheless, it is important to note that the results of this study do not advocate for the condemnation of digital technology in the modern age. There is scientific evidence that demonstrates the efficacy of educational apps in supporting preschool learning under adult guidance [102].

## 5. Conclusions

In conclusion, our findings highlight the potential risks associated with parental phubbing in the emergence of behavioral problems among preschool-aged children, while also highlighting parental mindfulness as both a protective factor against technology-based disruptions in parent–child interactions and a promising target for intervention.

By enhancing present-moment awareness and emotional regulation, mindful parenting may reduce the likelihood of parental phubbing, thereby promoting more responsive and engaged caregiving.

The present study is subject to the limitations inherent in research of this nature. In particular, the cross-sectional design of the study precludes the formulation of conclusions that would be considered causal statements. It is therefore recommended that future studies examine the same relationship through the medium of longitudinal studies.

Moreover, the data pertaining to the measures were collected via self-report questionnaires, a method known to prompt socially desirable responses. It is recommended that future studies concentrate on the ecological observation of phubbing behavior within a domestic context.

Future research should explore longitudinal effects and examine potential mediating variables—such as parental stress, attachment styles, and coping strategies of both children and parents—to better understand the mechanisms at play. Ultimately, integrating mindfulness-based strategies into parenting programs could offer a promising avenue for strengthening parent–child relationships and supporting positive developmental outcomes across early childhood. Although further research is needed, understanding how parental mindfulness influences phubbing can provide valuable insights for early interventions aimed at reducing problem behaviors in young children. Therefore, addressing this early on may benefit both parents and children, fostering healthier family dynamics and child development.

## Data Availability

Datasets generated and/or analyzed during the study are available from the first author on reasonable request.

## References

[1] Chotpitayasunondh V., Douglas K.M. (2016). How “Phubbing” Becomes the Norm: The Antecedents and Consequences of Snubbing via Smartphone. Comput. Hum. Behav..

[2] Chotpitayasunondh V., Douglas K.M. (2018). The Effects of “Phubbing” on Social Interaction. J. Appl. Soc. Psychol..

[3] Karadaǧ E., Tosuntaş Ş.B., Erzen E., Duru P., Bostan N., Şahin B.M., Çulha I., Babadaǧ B. (2015). Determinants of Phubbing, Which Is the Sum of Many Virtual Addictions: A Structural Equation Model. J. Behav. Addict..

[4] Roberts J.A., David M.E. (2017). Put down Your Phone and Listen to Me: How Boss Phubbing Undermines the Psychological Conditions Necessary for Employee Engagement. Comput. Hum. Behav..

[5] Roberts J.A., David M.E. (2016). My Life Has Become a Major Distraction from My Cell Phone: Partner Phubbing and Relationship Satisfaction among Romantic Partners. Comput. Hum. Behav..

[6] Ugur N.G., Koc T. (2015). Time for Digital Detox: Misuse of Mobile Technology and Phubbing. Procedia Soc. Behav. Sci..

[7] Davey S., Davey A., Raghav S.K., Singh J.V., Singh N., Blachnio A., Przepiórkaa A. (2018). Predictors and Consequences of “Phubbing” among Adolescents and Youth in India: An Impact Evaluation Study. J. Fam. Community Med..

[8] Stockdale L.A., Coyne S.M., Padilla-Walker L.M. (2018). Parent and Child Technoference and Socioemotional Behavioral Outcomes: A Nationally Representative Study of 10- to 20-Year-Old Adolescents. Comput. Hum. Behav..

[9] McDaniel B.T., Galovan A.M., Cravens J.D., Drouin M. (2018). “Technoference” and Implications for Mothers’ and Fathers’ Couple and Coparenting Relationship Quality. Comput. Hum. Behav..

[10] Braune-Krickau K., Schneebeli L., Pehlke-Milde J., Gemperle M., Koch R., von Wyl A. (2021). Smartphones in the Nursery: Parental Smartphone Use and Parental Sensitivity and Responsiveness within Parent–Child Interaction in Early Childhood (0–5 Years): A Scoping Review. Infant Ment. Health. J..

[11] Kushlev K., Dunn E.W. (2019). Smartphones Distract Parents from Cultivating Feelings of Connection When Spending Time with Their Children. J. Soc. Pers. Relat..

[12] Li J., Jiang Y., Xiao B., Wang J., Zhang Q., Zhang W., Li Y. (2024). Validation of a Revised Parental Phubbing Scale for Parents of Young Children in China. Early Child. Dev. Care..

[13] Wang X., Qiao Y., Li W., Lei L. (2022). Parental Phubbing and Children’s Social Withdrawal and Aggression: A Moderated Mediation Model of Parenting Behaviors and Parents’ Gender. J. Interpers. Violence.

[14] McDaniel B.T., Radesky J.S. (2018). Technoference: Parent Distraction with Technology and Associations with Child Behavior Problems. Child Dev..

[15] Boles R.E., Roberts M.C. (2008). Supervising Children during Parental Distractions. J. Pediatr. Psychol..

[16] Moran K. (2010). Watching Parents, Watching Kids: Water Safety Supervision of Young Children at the Beach. Int. J. Aquat. Res. Educ..

[17] Palsson C. (2014). That Smarts!: Smartphones and Child Injuries. https://citeseerx.ist.psu.edu/document?repid=rep1&type=pdf&doi=3c11291c64570e73e9fb7c1b94c0d5d206a513be.

[18] Steiner-Adair C., Barker T.H. (2013). The Big Disconnect—Procting Childhood and Family Relationships in the Digital Age.

[19] Kildare C.A., Middlemiss W. (2017). Impact of Parents Mobile Device Use on Parent-Child Interaction: A Literature Review. Comput. Hum. Behav..

[20] Błachnio A., Przepiórka A., Gorbaniuk O., Kot P., Chmielik M., Sobol M. (2023). Look at Me, I Am Here! A Diary Study on Parental Phubbing and Children’s Delay of Gratification. Annu. Rev. CyberTherapy Telemed..

[21] Bitar Z., Hallit S., Khansa W., Obeid S. (2021). Phubbing and Temperaments among Young Lebanese Adults: The Mediating Effect of Self-Esteem and Emotional Intelligence. BMC Psychol..

[22] Hong W., Liu R.D., Ding Y., Oei T.P., Zhen R., Jiang S. (2019). Parents’ Phubbing and Problematic Mobile Phone Use: The Roles of the Parent-Child Relationship and Children’s Self-Esteem. Cyberpsychology Behav. Soc. Netw..

[23] McDaniel B.T. (2019). Parent Distraction with Phones, Reasons for Use, and Impacts on Parenting and Child Outcomes: A Review of the Emerging Research. Hum. Behav. Emerg. Technol..

[24] McDaniel B.T., Coyne S.M. (2016). Technology Interference in the Parenting of Young Children: Implications for Mothers’ Perceptions of Coparenting. Soc. Sci. J..

[25] Xie X., Chen W., Zhu X., He D. (2019). Parents’ Phubbing Increases Adolescents’ Mobile Phone Addiction: Roles of Parent-Child Attachment, Deviant Peers, and Gender. Child. Youth Serv. Rev..

[26] Kabat-Zinn J. (2015). Mindfulness. Mindfulness.

[27] Bishop S.R., Lau M., Shapiro S., Carlson L., Anderson N.D., Carmody J., Segal Z.V., Abbey S., Speca M., Velting D. (2004). Mindfulness: A Proposed Operational Definition. Clin. Psychol. Sci. Pract..

[28] Anshari M., Almunawar M.N., Shahrill M., Wicaksono D.K., Huda M. (2017). Smartphones Usage in the Classrooms: Learning Aid or Interference?. Educ. Inf. Technol..

[29] Przybylski A.K., Weinstein N. (2013). Can You Connect with Me Now? How the Presence of Mobile Communication Technology Influences Face-to-Face Conversation Quality. J. Soc. Pers. Relat..

[30] Tanil C.T., Yong M.H. (2020). Mobile Phones: The Effect of Its Presence on Learning and Memory. PLoS ONE.

[31] Vanden Abeele M.M.P., Antheunis M.L., Schouten A.P. (2016). The Effect of Mobile Messaging during a Conversation on Impression Formation and Interaction Quality. Comput. Hum. Behav..

[32] Shao L., Dong Y., Feng J.X., Zhang D.H. (2020). The Relationship between Parent Phubbing and Aggression among Adolescents: The Role of Ostracism and Interpersonal Sensitivity. Psychology.

[33] Karadağ E., Tosuntaş Ş.B., Erzen E., Duru P., Bostan N., Mızrak Şahin B., Çulha İ., Babadağ B. (2016). The Virtual World’s Current Addiction: Phubbing. Addicta Turk. J. Addict..

[34] Dwyer R.J., Kushlev K., Dunn E.W. (2018). Smartphone Use Undermines Enjoyment of Face-to-Face Social Interactions. J. Exp. Soc. Psychol..

[35] Arpaci I. (2021). Relationships Between Early Maladaptive Schemas and Smartphone Addiction: The Moderating Role of Mindfulness. Int. J. Ment. Health Addict..

[36] Regan T., Harris B., Van Loon M., Nanavaty N., Schueler J., Engler S., Fields S.A. (2020). Does Mindfulness Reduce the Effects of Risk Factors for Problematic Smartphone Use? Comparing Frequency of Use versus Self-Reported Addiction. Addict. Behav..

[37] Montag C., Walla P. (2016). Carpe Diem Instead of Losing Your Social Mind: Beyond Digital Addiction and Why We All Suffer from Digital Overuse. Cogent Psychol..

[38] Liu Q.Q., Zhang D.J., Yang X.J., Zhang C.Y., Fan C.Y., Zhou Z.K. (2018). Perceived Stress and Mobile Phone Addiction in Chinese Adolescents: A Moderated Mediation Model. Comput. Hum. Behav..

[39] Song W.J., Park J.W. (2019). The Influence of Stress on Internet Addiction: Mediating Effects of Self-Control and Mindfulness. Int. J. Ment. Health Addict..

[40] Hölzel B.K., Carmody J., Vangel M., Congleton C., Yerramsetti S.M., Gard T., Lazar S.W. (2011). Mindfulness Practice Leads to Increases in Regional Brain Gray Matter Density. Psychiatry Res. Neuroimaging.

[41] Oulasvirta A., Rattenbury T., Ma L., Raita E. (2012). Habits Make Smartphone Use More Pervasive. Pers. Ubiquitous Comput..

[42] Van Deursen A.J.A.M., Bolle C.L., Hegner S.M., Kommers P.A.M. (2015). Modeling Habitual and Addictive Smartphone Behavior: The Role of Smartphone Usage Types, Emotional Intelligence, Social Stress, Self-Regulation, Age, and Gender. Comput. Hum. Behav..

[43] Bradford B. No Time to Think: The Impact of Smartphone Technology on Mindfulness and Reflection. https://www.academia.edu/36251280/No_time_to_think_The_impact_of_smartphone_technology_on_mindfulness_and_reflection.

[44] Calvete E., Gámez-Guadix M., Cortazar N. (2017). Mindfulness Facets and Problematic Internet Use: A Six-Month Longitudinal Study. Addict. Behav..

[45] Faruk C. (2023). The Relationship Between Partner Phubbing and Life Satisfaction: The Mediating Role of Relationship Satisfaction and Perceived Romantic Relationship Quality. Psychol. Rep..

[46] Burt K.B., Obradović J., Long J.D., Masten A.S. (2008). The Interplay of Social Competence and Psychopathology over 20 Years: Testing Transactional and Cascade Models. Child Dev..

[47] Achenbach T.M., Rescorla L. (2021). Manual for the ASEBA School-Age Forms & Profiles: An Integrated System of Multi-Informant Assessment.

[48] Achenbach T.M. (2009). The Achenbach System of Empirically Based Assessment (ASEBA): Development, Findings, Theory, and Applications.

[49] Baumrind D. (1991). The Influence of Parenting Style on Adolescent Competence and Substance Use. J. Early Adolesc..

[50] Wang X., Wang W., Qiao Y., Gao L., Yang J., Wang P. (2022). Parental Phubbing and Adolescents’ Cyberbullying Perpetration: A Moderated Mediation Model of Moral Disengagement and Online Disinhibition. J. Interpers. Violence.

[51] Solecki S. (2022). The Phubbing Phenomenon: The Impact on Parent-Child Relationships. J. Pediatr. Nurs..

[52] Wang X., Gao L., Yang J., Zhao F., Wang P. (2020). Parental Phubbing and Adolescents’ Depressive Symptoms: Self-Esteem and Perceived Social Support as Moderators. J. Youth Adolesc..

[53] McDaniel B.T., Radesky J.S. (2018). Technoference: Longitudinal Associations between Parent Technology Use, Parenting Stress, and Child Behavior Problems. Pediatr. Res..

[54] Rubin K.H., Coplan R.J., Bowker J.C. (2009). Social Withdrawal in Childhood. Annu. Rev. Psychol..

[55] Zarra-Nezhad M., Kiuru N., Aunola K., Zarra-Nezhad M., Ahonen T., Poikkeus A.M., Lerkkanen M.K., Nurmi J.E. (2014). Social Withdrawal in Children Moderates the Association between Parenting Styles and the Children’s Own Socioemotional Development. J. Child Psychol. Psychiatry.

[56] Liu J. (2004). Childhood Externalizing Behavior: Theory and Implications. J. Child Adolesc. Psychiatr. Nurs..

[57] Bongers I.L., Koot H.M., Van Der Ende J., Verhulst F.C. (2008). Predicting Young Adult Social Functioning from Developmental Trajectories of Externalizing Behaviour. Psychol. Med..

[58] Radesky J.S., Silverstein M., Zuckerman B., Christakis D.A. (2014). Infant Self-Regulation and Early Childhood Media Exposure. Pediatrics.

[59] Eisenberg N., Spinrad T.L., Knafo-Noam A., Lamb M.E. (2015). Prosocial Development. Handbook of Child Psychology and Developmental Science. Vol. 3: Socioemotional Processes.

[60] Mieloo C., Raat H., van Oort F., Bevaart F., Vogel I., Donker M., Jansen W. (2012). Validity and Reliability of the Strengths and Difficulties Questionnaire in 5–6 Year Olds: Differences by Gender or by Parental Education?. PLoS ONE.

[61] Maurice-Stam H., Haverman L., Splinter A., van Oers H.A., Schepers S.A., Grootenhuis M.A. (2018). Dutch Norms for the Strengths and Difficulties Questionnaire (SDQ)—Parent Form for Children Aged 2–18 years. Health Qual. Life Outcomes.

[62] Daniel E., Madigan S., Jenkins J. (2016). Paternal and Maternal Warmth and the Development of Prosociality among Preschoolers. J. Fam. Psychol..

[63] Duncan L.G., Coatsworth J.D., Greenberg M.T. (2009). A Model of Mindful Parenting: Implications for Parent-Child Relationships and Prevention Research. Clin. Child Fam. Psychol. Rev..

[64] Coatsworth J.D., Duncan L.G., Greenberg M.T., Nix R.L. (2010). Changing Parent’s Mindfulness, Child Management Skills and Relationship Quality with Their Youth: Results from a Randomized Pilot Intervention Trial. J. Child Fam. Stud..

[65] Coatsworth J.D., Duncan L.G., Nix R.L., Greenberg M.T., Gayles J.G., Bamberger K.T., Berrena E., Demi M.A. (2015). Integrating Mindfulness with Parent Training: Effects of the Mindfulness-Enhanced Strengthening Families Program. Dev. Psychol..

[66] Medeiros C., Gouveia M.J., Canavarro M.C., Moreira H. (2016). The Indirect Effect of the Mindful Parenting of Mothers and Fathers on the Child’s Perceived Well-Being Through the Child’s Attachment to Parents. Mindfulness.

[67] Bröning S., Brandt M. (2022). ”Mindful Parenting”—Achtsamkeit in Der Eltern-Kind-Beziehung. Z Kinder Jugendpsychiatr Psychother.

[68] Neece C.L., Chan N., Klein K., Roberts L., Fenning R.M. (2019). Mindfulness-Based Stress Reduction for Parents of Children with Developmental Delays: Understanding the Experiences of Latino Families. Mindfulness.

[69] Singh N.N., Lancioni G.E., Winton A.S.W., Fisher B.C., Wahler R.G., McAleavey K., Singh J., Sabaawi M. (2006). Mindful Parenting Decreases Aggression, Noncompliance, and Self-Injury in Children with Autism. J. Emot. Behav. Disord..

[70] Park Y.R., Nix R.L., Duncan L.G., Coatsworth J.D., Greenberg M.T. (2020). Unfolding Relations among Mindful Parenting, Recurrent Conflict, and Adolescents’ Externalizing and Internalizing Problems. Fam. Process.

[71] Yang W., Deng J., Wang Y. (2022). The Association Between Mindful Parenting and Adolescent Internalizing and Externalizing Problems: The Role of Mother–Child Communication. Child Psychiatry Hum. Dev..

[72] Donovan M.O., Pickard J.A., Herbert J.S., Barkus E. (2022). Mindful Parent Training for Parents of Children Aged 3–12 Years with Behavioral Problems: A Scoping Review. Mindfulness.

[73] Townshend K., Jordan Z., Stephenson M., Tsey K. (2016). The Effectiveness of Mindful Parenting Programs in Promoting Parents’ and Children’s Wellbeing: A Systematic Review. JBI Database Syst. Rev. Implement. Rep..

[74] Bögels S., Hoogstad B., Van Dun L., De Schutter S., Restifo K. (2008). Mindfulness Training for Adolescents with Externalizing Disorders and Their Parents. Behav. Cogn. Psychother..

[75] Geurtzen N., Scholte R.H.J., Engels R.C.M.E., Tak Y.R., van Zundert R.M.P. (2015). Association Between Mindful Parenting and Adolescents’ Internalizing Problems: Non-Judgmental Acceptance of Parenting as Core Element. J. Child Fam. Stud..

[76] Zou Z., Zeng X. (2024). The association of mindful parenting with preschool children’s problem behaviors: Parental phubbing as a mediator. Psychologia.

[77] Huang J., Chen X., Wu D., Liu R., Xie S., Chi X. (2025). Mindful Parenting and Preschoolers’ Digital Overuse: Exploring the Roles of Executive Function and Gender Differences. Child. Youth Serv. Rev..

[78] Schoemann A.M., Boulton A.J., Short S.D. (2017). Determining Power and Sample Size for Simple and Complex Mediation Models. Soc. Psychol. Personal. Sci..

[79] Chotpitayasunondh V., Douglas K.M. (2018). Measuring Phone Snubbing Behavior: Development and Validation of the Generic Scale of Phubbing (GSP) and the Generic Scale of Being Phubbed (GSBP). Comput. Hum. Behav..

[80] Brown K.W., Ryan R.M. (2003). Mindful Attention Awereness Scale. J. Personal. Soc. Psychol..

[81] Veneziani C.A., Voci A. (2015). The Italian Adaptation of the Mindful Awareness Attention Scale and Its Relation with Individual Differences and Quality of Life Indexes. Mindfulness.

[82] Goodman R. (1994). A Modified Version of the Rutter Parent Questionnaire Including Extra Items on Children’s Strengths: A Research Note. J. Child Psychol. Psychiatry.

[83] Rutter M. (1967). A children’s behaviour questionnaire for completion by teachers: Preliminary findings. J. Child Psychol. Psychiatry.

[84] Tobia V., Gabriele M.A., Marzocchi G.M. (2013). The Italian Version of the Strengths and Difficulties Questionnaire (SDQ)-Teacher: Psychometric Properties. J. Psychoeduc. Assess..

[85] Marzocchi G.M., Pietro M.D., Vio C., Bassi E., Filoramo G., Salmaso A. (2003). Il Questionario SDQ. Strengtht and Difficulties Questionnaire: Uno Strumento per Valutare Difficoltà Comportamentali Ed Emotive in Età Evolutiva. Difficoltà Apprendimento.

[86] JASP Team (2004). JASP(Version 0.19.3) 2024.

[87] R Development Core Team (2024). RStudio (Version 2023.03.1) 2024.

[88] Bögels S.M., Lehtonen A., Restifo K. (2010). Mindful Parenting in Mental Health Care. Mindfulness.

[89] Parent J., McKee L.G., Anton M., Gonzalez M., Jones D.J., Forehand R. (2016). Mindfulness in Parenting and Coparenting. Mindfulness.

[90] Van der Oord S., Bögels S.M., Peijnenburg D. (2012). The Effectiveness of Mindfulness Training for Children with ADHD and Mindful Parenting for Their Parents. J. Child Fam. Stud..

[91] Turpyn C.C., Chaplin T.M. (2016). Mindful Parenting and Parents’ Emotion Expression: Effects on Adolescent Risk Behaviors. Mindfulness.

[92] Saral B., Acar I.H. (2021). Preschool Children’s Social Competence: The Roles of Parent–Child, Parent–Parent, and Teacher–Child Relationships. Eur. Early Child. Educ. Res. J..

[93] Lv H., Ye W., Chen S., Zhang H., Wang R. (2022). The Effect of Mother Phubbing on Young Children’s Emotional and Behavioral Problems: A Moderated Mediation Model of Mother–Child Attachment and Parenting Stress. Int. J. Environ. Res. Public Health.

[94] Sundqvist A., Heimann M., Koch F.S. (2020). Relationship between Family Technoference and Behavior Problems in Children Aged 4–5 Years. Cyberpsychol. Behav. Soc. Netw..

[95] Carson V., Kuzik N. (2021). The Association between Parent–Child Technology Interference and Cognitive and Social–Emotional Development in Preschool-Aged Children. Child Care Health Dev..

[96] Niu G., Yao L., Wu L., Tian Y., Xu L., Sun X. (2020). Parental Phubbing and Adolescent Problematic Mobile Phone Use: The Role of Parent-Child Relationship and Self-Control. Child. Youth Serv. Rev..

[97] Xu C., Xie X. (2023). Put down the Phone and Accompany Me: How Parental Phubbing Undermines Prosocial Behavior of Early Adolescents. Child Youth Serv. Rev..

[98] Kakhki Z.B., Mashhadi A., Yazdi S.A.A., Saleh S. (2022). The Effect of Mindful Parenting Training on Parent–Child Interactions, Parenting Stress, and Cognitive Emotion Regulation in Mothers of Preschool Children. J. Child Fam. Stud..

[99] Lippold M.A., Duncan L.G., Coatsworth J.D., Nix R.L., Greenberg M.T. (2015). Understanding How Mindful Parenting May Be Linked to Mother–Adolescent Communication. J. Youth Adolesc..

[100] Dumas J.E. (2005). Mindfulness-Based Parent Training: Strategies to Lessen the Grip of Automaticity in Families with Disruptive Children. J. Clin. Child Adolesc. Psychol..

[101] Potharst E.S., Leyland A., Colonnesi C., Veringa I.K., Salvadori E.A., Jakschik M., Bögels S.M., Zeegers M.A.J. (2021). Does Mothers’ Self-Reported Mindful Parenting Relate to the Observed Quality of Parenting Behavior and Mother-Child Interaction?. Mindfulness.

[102] Perini N., Porru A., Moeller K., Jay T., Sella F. Number Express: A Digital Game to Improve Early Numeracy. Proceedings of the 17th European Conference on Games Based Learning, ECGBL 2023.

